# Selective DNA and Protein Isolation From Marine Macrophyte Surfaces

**DOI:** 10.3389/fmicb.2021.665999

**Published:** 2021-05-24

**Authors:** Marino Korlević, Marsej Markovski, Zihao Zhao, Gerhard J. Herndl, Mirjana Najdek

**Affiliations:** ^1^Center for Marine Research, Ruđer Bošković Institute, Rovinj, Croatia; ^2^Department of Functional and Evolutionary Ecology, University of Vienna, Vienna, Austria; ^3^Department of Marine Microbiology and Biogeochemistry, Royal Netherlands Institute for Sea Research (NIOZ), Utrecht University, Den Burg, Netherlands; ^4^Vienna Metabolomics Center, University of Vienna, Vienna, Austria

**Keywords:** selective isolation, DNA, proteins, marine macrophytes, *Cymodocea nodosa*, *Caulerpa cylindracea*

## Abstract

Studies of unculturable microbes often combine methods, such as 16S rRNA sequencing, metagenomics, and metaproteomics. To apply these techniques to the microbial community inhabiting the surfaces of marine macrophytes, it is advisable to perform a selective DNA and protein isolation prior to the analysis to avoid biases due to the host material being present in high quantities. Two protocols for DNA and protein isolation were adapted for selective extractions of DNA and proteins from epiphytic communities inhabiting the surfaces of two marine macrophytes, the seagrass *Cymodocea nodosa* and the macroalga *Caulerpa cylindracea*. Protocols showed an almost complete removal of the epiphytic community regardless of the sampling season, station, settlement, or host species. The obtained DNA was suitable for metagenomic and 16S rRNA sequencing, while isolated proteins could be identified by mass spectrometry. Low presence of host DNA and proteins in the samples indicated a high specificity of the protocols. The procedures are based on universally available laboratory chemicals making the protocols widely applicable. Taken together, the adapted protocols ensure an almost complete removal of the macrophyte epiphytic community. The procedures are selective for microbes inhabiting macrophyte surfaces and provide DNA and proteins applicable in 16S rRNA sequencing, metagenomics, and metaproteomics.

## Introduction

Surfaces of marine macrophytes are colonized by a diverse microbial community whose structure and function are poorly understood (Egan et al., 2013). As <1% of all prokaryotic species are culturable, molecular methods, such as 16S rRNA sequencing, metagenomics, and metaproteomics are indispensable to study these organisms (Amann et al., 1995; Su et al., 2012). Applying these techniques requires an initial isolation step with the purpose of obtaining high-quality DNA and proteins.

Biological material (i.e., proteins and DNA) from pelagic microbial communities is usually isolated by collecting cells onto filters and subsequently isolating the target organisms or communities (Gilbert et al., 2009). If a specific microbial size fraction is aimed, sequential filtration is applied (Massana et al., 1997; Andersson et al., 2010). In contrast, obtaining microorganisms associated to surfaces require either a cell detachment procedure prior to isolation or the host material is co-extracted with the target material. Methods for separating microbial cells from the host include shaking of host tissue (Gross et al., 2003; Nõges et al., 2010), scraping of macrophyte surfaces (Uku et al., 2007), or applying ultrasonication (Weidner et al., 1996; Cai et al., 2014). It was shown that shaking alone is not sufficient to remove microbial cells from surfaces, at least not from plant root surfaces (Richter-Heitmann et al., 2016). Manual separation methods, such as scraping and brushing are time consuming and subjective, as the detachment efficiency depends on host tissue and the person performing the procedure (Cai et al., 2014). Ultrasonication was proposed as an alternative method as it is providing better results in terms of detachment efficiency (Cai et al., 2014; Richter-Heitmann et al., 2016). The downside of this procedure is that complete cell removal is still not obtained and tissue disruption was observed especially after the application of probe ultrasonication (Richter-Heitmann et al., 2016). An alternative to these cell detachment procedures is the isolation of target epiphytic compounds together with host material (Staufenberger et al., 2008; Jiang et al., 2015). This procedure can lead to problems in the following processing steps, such as mitochondrial and chloroplast 16S rRNA sequence contaminations from the host (Longford et al., 2007; Staufenberger et al., 2008). In addition, when performing metagenomics and metaproteomics host material can cause biased results toward more abundant host DNA and proteins.

An alternative to these procedures is a direct isolation of the target material by incubating macrophyte tissues in an extraction buffer. After the incubation, the undisrupted host tissue is removed followed by the isolation procedure, omitting host material contaminations. To our knowledge, the only procedure describing a direct and selective epiphytic DNA isolation from the surfaces of marine macrophytes was described by Burke et al. (2009). In contrast to previously described methods, this protocol enables an almost complete removal of the surface community. It was used for 16S rRNA gene clone library construction (Burke et al., 2011b) and metagenome sequencing (Burke et al., 2011a). This method, although providing a selective isolation procedure, uses a rapid multi-enzyme cleaner (3M) that is not available worldwide and the chemical constituents are unknown (Burke et al., 2009). Also to our knowledge, no selective isolation protocol to perform (meta)proteomics of epiphytic communities associated with marine macrophytes has been developed yet.

In the present study, we adapted a protocol widely used for DNA isolation from filters (Massana et al., 1997) and a protocol used for protein isolation from soils (Chourey et al., 2010; Hultman et al., 2015). These two adapted methods allowed for a selective extraction of DNA and proteins from epiphytic communities inhabiting the surfaces of two marine macrophytes, the seagrass *Cymodocea nodosa*, and the macroalga *Caulerpa cylindracea*. In addition, we tested the removal efficiency of the protocol and the suitability of obtained DNA and proteins for 16S rRNA sequencing, metagenomics, and metaproteomics.

## Materials and Methods

### Sampling

Leaves of *C. nodosa* were sampled in a *C. nodosa* meadow in the Bay of Saline, northern Adriatic Sea (45°7′5″ N, 13°37′20″ E) and in a *C. nodosa* meadow invaded by *C. cylindracea* in the Bay of Funtana, northern Adriatic Sea (45°10′39″ N, 13°35′42″ E). Thalli of *C. cylindracea* were sampled in the same *C. nodosa* invaded meadow in the Bay of Funtana and at a locality of only *C. cylindracea* located in the proximity of the invaded meadow. Leaves and thalli for 16S rRNA analysis, metagenomics, and metaproteomics were collected in two contrasting seasons, on December 4, 2017 (16S rRNA analysis and metaproteomics), December 14, 2017 (metagenomics), and June 18, 2018 (16S rRNA analysis, metagenomics, and metaproteomics). During spring 2018, the *C. nodosa* meadow in the Bay of Saline decayed to an extent that no leaves could be retrieved (Najdek et al., 2020). In addition, as not enough DNA for both metagenomic and 16S RNA analysis were obtained during the sampling on December 4, 2017, an additional sampling on December 14, 2017 was carried out in the Bay of Funtana. Leaves and thalli were collected by diving and transported to the laboratory in containers placed on ice and filled with seawater from the site. Upon arrival to the laboratory, *C. nodosa* leaves were cut into sections of 1–2 cm, while *C. cylindracea* thalli were cut into 5–8 cm long sections. Leaves and thalli were washed three times with sterile artificial seawater (ASW) to remove loosely attached microbial cells.

### DNA Isolation

The DNA was isolated according to the protocol for isolation from filters described in Massana et al. (1997). This protocol was modified and adapted for microbial DNA isolation from macrophyte surfaces as described below. Five milliliter of lysis buffer (40 mm EDTA, 50 mm Tris-HCl, 0.75 m sucrose; pH 8.3) was added to 1 g wet weight of leaves or 2 g wet weight of thalli. For every sample, duplicate isolations were performed. Lysozyme was added (final concentration 1 mg ml-1) and the mixture was incubated at 37°C for 30 min. Subsequently, proteinase K (final concentration 0.5 mg ml-1) and SDS (final concentration 1%) were added and the samples were incubated at 55°C for 2 h. Following the incubation, tubes were vortexed for 10 min and the mixture containing lysed epiphytic cells was separated from host leaves or thalli by transferring the solution into a clean tube. The lysate was extracted twice with a mixture of phenol:chloroform:isoamyl alcohol (25:24:1; pH 8) and once with chloroform:isoamyl alcohol (24:1). After each addition of an organic solvent mixture, tubes were slightly vortexed and centrifuged at 4,500 × g for 10 min. Following each centrifugation, the aqueous phases were retrieved. After the final extraction, 1/10 of chilled 3 m sodium acetate (pH 5.2) was added. DNA was precipitated by adding 1 volume of chilled isopropanol, incubating the mixtures overnight at -20°C and centrifuging at 16,000 × g and 4°C for 20 min. The pellet was washed twice with 1 ml of chilled 70% ethanol and centrifuged after each washing step at 20,000 × g and 4°C for 10 min. After the first washing step, duplicate pellets from the same sample were pooled and transferred to a clean 1.5 ml tube. The dried pellet was re-suspended in 100 μl of deionized water.

### Illumina 16S rRNA Sequencing

An aliquot of isolated DNA was treated with RNase A (final concentration 200 μg ml-1) for 2 h at 37°C. The DNA concentration was determined using the Quant-iT PicoGreen dsDNA Assay Kit (Invitrogen, USA) according to the manufacturer's instructions and diluted to 1 ng μl-1. The V4 region of the 16S rRNA gene was amplified using a two-step PCR procedure. In the first PCR, the 515F (5′-GTGYCAGCMGCCGCGGTAA-3′) and 806R (5′-GGACTACNVGGGTWTCTAAT-3′) primers from the Earth Microbiome Project (https://earthmicrobiome.org/protocols-and-standards/16s/) were used to amplify the target region (Caporaso et al., 2012; Apprill et al., 2015; Parada et al., 2016). These primers contained on their 5′ end a tagged sequence. Each sample was amplified in four parallel 25 μl reactions, in which each contained 1 × Q5 reaction buffer, 0.2 mm dNTPmix, 0.7 mg ml-1 BSA (bovine serum albumin), 0.2 μm forward and reverse primers, 0.5 U of Q5 High-Fidelity DNA Polymerase (New England Biolabs, USA), and 5 ng of DNA template. Cycling conditions were as follows: initial denaturation at 94°C for 3 min, 20 cycles of denaturation at 94°C for 45 s, annealing at 50°C for 60 s and elongation at 72°C for 90 s, finalized by an elongation step at 72°C for 10 min. The four parallel reactions volumes were pooled and PCR products were purified using the GeneJET PCR Purification Kit (Thermo Fisher Scientific, USA) according to the manufacturer's instructions and following the protocol that included isopropanol addition for better small DNA fragment yield. The column was eluted in 30 μl of deionized water. Purified PCR products were sent for Illumina MiSeq sequencing (2 ×250 bp) at IMGM Laboratories, Martinsried, Germany. Before sequencing at IMGM, the second PCR amplification of the two-step PCR procedure was performed using primers targeting the tagged region incorporated in the first PCR. In addition, these primers contained adapter and sample-specific index sequences. The second PCR was carried out for eight cycles. Beside samples, a positive and negative control were sequenced. A negative control was composed of four parallel PCR reactions without DNA template, while for a positive control a mock community composed of evenly mixed DNA material originating from 20 bacterial strains (ATCC MSA-1002, ATCC, USA) was used. Partial 16S rRNA sequences obtained in this study have been deposited in the European Nucleotide Archive (ENA) at EMBL-EBI under accession numbers SAMEA6786270, SAMEA6648792–SAMEA6648794, SAMEA6648809–SAMEA6648811, and SAMEA6648824.

Obtained sequences were analyzed on the computer cluster Isabella (University Computing Center, University of Zagreb) using mothur (version 1.43.0) (Schloss et al., 2009) according to the MiSeq Standard Operating Procedure (MiSeq SOP; https://mothur.org/wiki/MiSeq_SOP) (Kozich et al., 2013) and recommendations given from the Riffomonas project to enhance data reproducibility (http://www.riffomonas.org/). For alignment and classification of sequences, the SILVA SSU Ref NR 99 database (release 138; http://www.arb-silva.de) was used (Quast et al., 2013; Yilmaz et al., 2014). Sequences classified as chloroplasts by SILVA were exported and reclassified using mothur and the RDP (Ribosomal Database Project; http://rdp.cme.msu.edu/) training set (version 16) reference files adapted for mothur (Cole et al., 2014). In comparison to SILVA, RDP allows a more detailed classification of chloroplast sequences. Based on the ATCC MSA-1002 mock community included in the analysis, a sequencing error rate of 0.009% was determined, which is in line with previously reported values for next-generation sequencing data (Kozich et al., 2013; Schloss et al., 2016). In addition, the negative control processed together with the samples yielded only 2 sequences after sequence quality curation.

### Metagenomics

Four DNA samples were selected and sent on dry ice to IMGM Laboratories, Martinsried, Germany for metagenomic sequencing. DNA was purified using AMPure XP Beads (Beckman Coulter, USA) applying a bead:DNA ratio of 1:1 (v/v), quantified with a Qubit dsDNA HS Assay Kit (Thermo Fisher Scientific, USA) and check for integrity on a 1% agarose gel. Metagenomic sequencing libraries were generated from 300 ng of input DNA using a NEBNext Ultra II FS DNA Library Prep Kit for Illumina (New England Biolabs, USA) according to the manufacturer's instructions. Fragments were selected (500–700 bp) using AMPure XP Beads, PCR enriched for 3–5 cycles and quality controlled. Libraries generated from different DNA samples were pooled and sequenced on an Illumina NovaSeq 6000 sequencing system (2 ×250 bp).

Obtained sequences were analyzed on the Life Science Compute Cluster (LiSC) (CUBE–Computational Systems Biology, University of Vienna). Individual sequences were assembled using MEGAHIT (version 1.1.2) (Li et al., 2015) under default settings. Putative genes were predicted from contigs longer than 200 bp using Prodigal (version 2.6.3) (Hyatt et al., 2010) in metagenome mode (-p meta). Abundances of predicted genes were expressed as Reads Per Kilobase Million (RPKM) and calculated using the BWA algorithm (version 0.7.16a) (Li and Durbin, 2009). All predicted genes were functionally annotated using the eggNOG-mapper (Huerta-Cepas et al., 2017) and eggNOG database (version 5.0) (Huerta-Cepas et al., 2019). Sequence taxonomy classification was determined using the lowest common ancestor algorithm adapted from DIAMOND (version 0.8.36) (Buchfink et al., 2015) and by searching against the NCBI non-redundant database (NR). To determine the phylogeny, the top 10% hits with an e-value <1 ×10^−5^ were used (--top 10). Sequence renaming, coverage information computing, and metagenomic statistics calculations were performed using software tools from BBTools (https://jgi.doe.gov/data-and-tools/bbtools). Metagenomic sequences obtained in this study have been deposited in the European Nucleotide Archive (ENA) at EMBL-EBI under accession numbers SAMEA6648795, SAMEA6648797, SAMEA6648809, and SAMEA6648811.

### Protein Isolation

Proteins were isolated according to the protocol for protein isolation from soil described in Chourey et al. (2010) and modified by Hultman et al. (2015). This protocol was further modified and adapted for microbial protein isolation from macrophyte surfaces as described below. Twenty milliliters of protein extraction buffer (4% SDS, 100 mm Tris-HCl [pH 8.0]) was added to 5 g wet weight of leaves or 10 g wet weight of thalli. The mixture was incubated in boiling water for 5 min, vortexed for 10 min, and incubated again in boiling water for 5 min. After a brief vortex, the lysate was transferred to a clean tube separating the host leaves or thalli from the mixture containing lysed epiphytic cells. Dithiothreitol (DTT; final concentration 24 mm) was added and proteins were precipitated with chilled 100% trichloroacetic acid (TCA; final concentration 20%) overnight at -20°C. Precipitated proteins were centrifuged at 10,000 × g and 4°C for 40 min. The obtained protein pellet was washed three times with chilled acetone. During the first washing step, the pellet was transferred to a clean 1.5-ml tube. After each washing step, samples were centrifuged at 20,000 × g and 4°C for 5 min. Dried pellets were stored at -80°C until further analysis.

### Metaproteomics

Isolated proteins were whole trypsin digested using the FASP (filter-aided sample preparation) Protein Digestion Kit (Expedeon, UK) according to the manufacturer's instructions with small modifications (Wiśniewski et al., 2009). Prior to loading the solution onto the column, protein pellets were solubilized in urea sample buffer included in the kit amended with DTT (final concentration 100 mm) for 45 min at room temperature and centrifuged at 20,000 × g for 2–5 min at room temperature to remove larger particles. The first washing step after protein solution loading was repeated twice. In addition, the centrifugation steps were prolonged if the column was clogged. Trypsin digestion was performed on column filters at 37°C overnight for 18 h. The final filtrate containing peptides was acidified with 1% (final concentration) trifluoroacetic acid (TFA), freezed at -80°C, lyophilized, and sent to VIME–Vienna Metabolomics Center (University of Vienna) for metaproteomic analysis. Peptides were re-suspended in 1% (final concentration) TFA, desalted using the Pierce C18 Tips (Thermo Fisher Scientific, USA) according to the manufacturer's instructions, and sequenced on a Q Exactive Hybrid Quadrupole-Orbitrap Mass Spectrometer (Thermo Fisher Scientific). Obtained MS/MS spectra were searched against a protein database composed of combined sequenced metagenomes obtained in this study using SEQUEST-HT engines and validated with Percolator in Proteome Discoverer 2.1 (Thermo Fisher Scientific). The target-decoy approach was used to reduce the probability of false peptide identification. Results whose false discovery rate at the peptide level was <1 % were kept. For protein identification, a minimum of two peptides and one unique peptide were required. For protein quantification, a chromatographic peak area-based free quantitative method was applied.

### Data Processing and Visualization

Processing and visualization of 16S rRNA, metagenomic, and metaproteomic data were done using R (version 3.6.0) (R Core Team, 2019), package tidyverse (version 1.3.0) (Wickham et al., 2019), and multiple other packages (Neuwirth, 2014; Xie, 2014, 2015, 2019a,b; Wilke, 2018; Xie et al., 2018; Allaire et al., 2019; Zhu, 2019; Bengtsson, 2020). The detailed analysis procedure including the R Markdown file for this paper are available as a GitHub repository (https://github.com/MicrobesRovinj/Korlevic_SelectiveRemoval_FrontMicrobiol_2021).

### Confocal Microscopy

Host leaves and thalli from DNA and protein isolation steps were washed seven times in deionized water and fixed with formaldehyde (final concentration ~3%). In addition, non-treated leaves and thalli, washed three times in ASW to remove loosely attached microbial cells, were fixed in the same concentration of formaldehyde and used as a positive control. For long-term storage, fixed leaves and thalli were immersed in a mixture of phosphate-buffered saline (PBS) and ethanol (1:1) and stored at -20°C. Treated and untreated segments of leaves and thalli were stained in a 2 × solution of SYBR Green I and examined under a Leica TCS SP8 X FLIM confocal microscope (Leica Microsystems, Germany).

## Results

**Figure 1:**
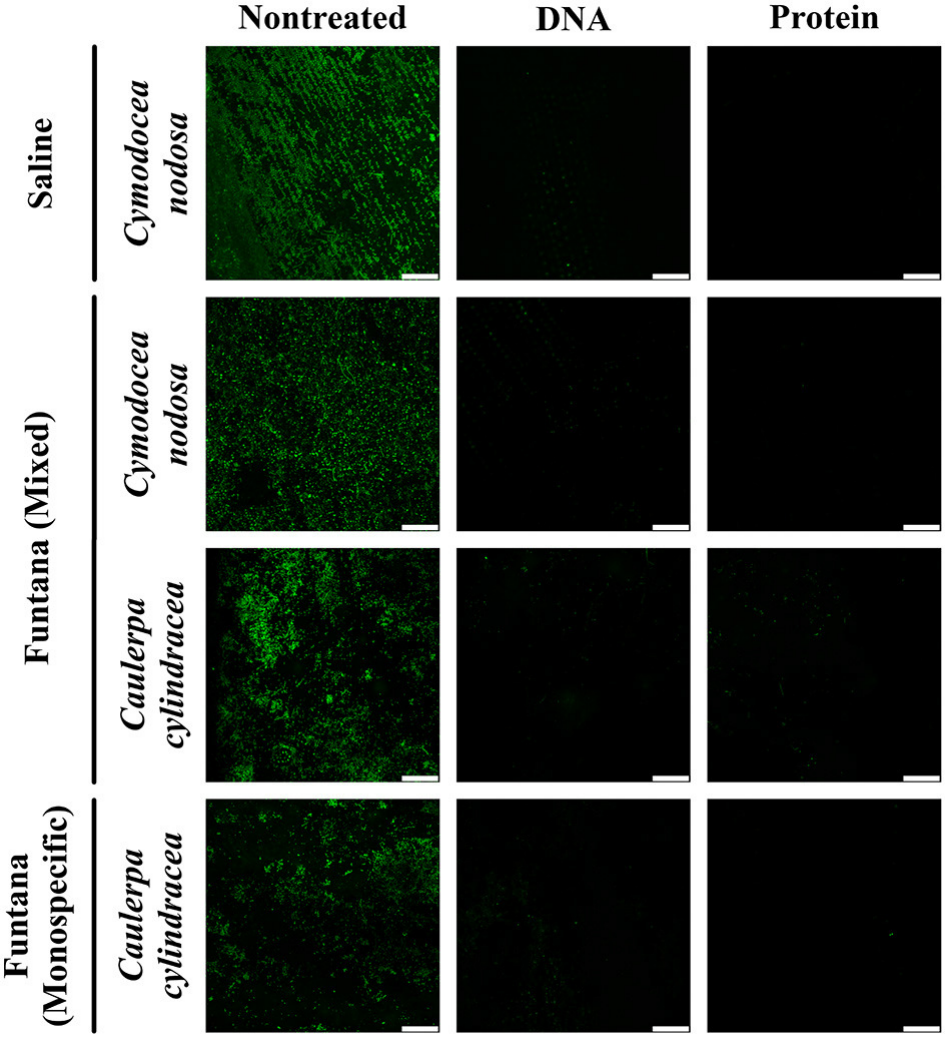
Confocal microscope images of *C. nodosa* and *C. cylindracea* surfaces from the Bay of Saline and the Bay of Funtana (mixed and monospecific settlements) sampled on December 4, 2017 and stained with SYBR Green I. Scale bar at all images is 60 μm.

**Figure 2:**
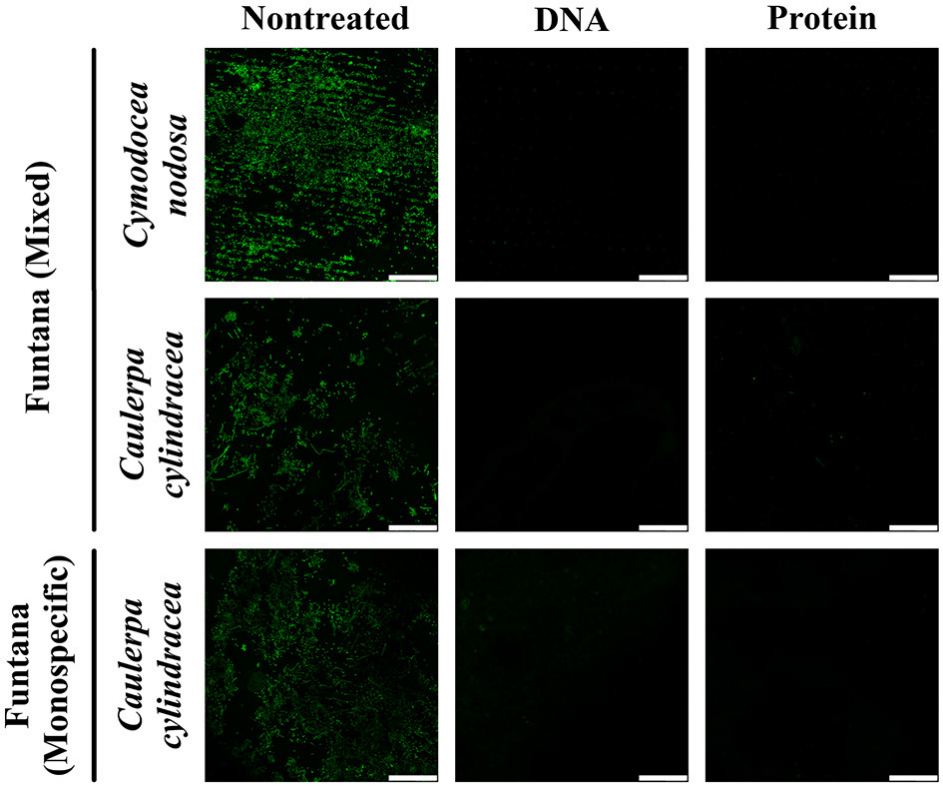
Confocal microscope images of *C. nodosa* and *C. cylindracea* surfaces from the Bay of Funtana (mixed and monospecific settlements) sampled on June 19, 2018 and stained with SYBR Green I. Scale bar at all images is 60 μm.

To assess the removal efficiency of the DNA and protein isolation procedures, leaves and thalli were examined under a confocal microscope before and after treatments were performed. The modified procedures resulted in an almost complete removal of the surface community of both, *C. nodosa* and *C. cylindracea*. In addition, a similar removal efficiency was observed for communities sampled in contrasting months, December 2017 (Figure 1) and June 2018 (Figure 2). Also, no effect of station, settlement, or isolation procedure (DNA or protein) on the removal efficiency was observed (Figures 1, 2).

**Figure 3:**
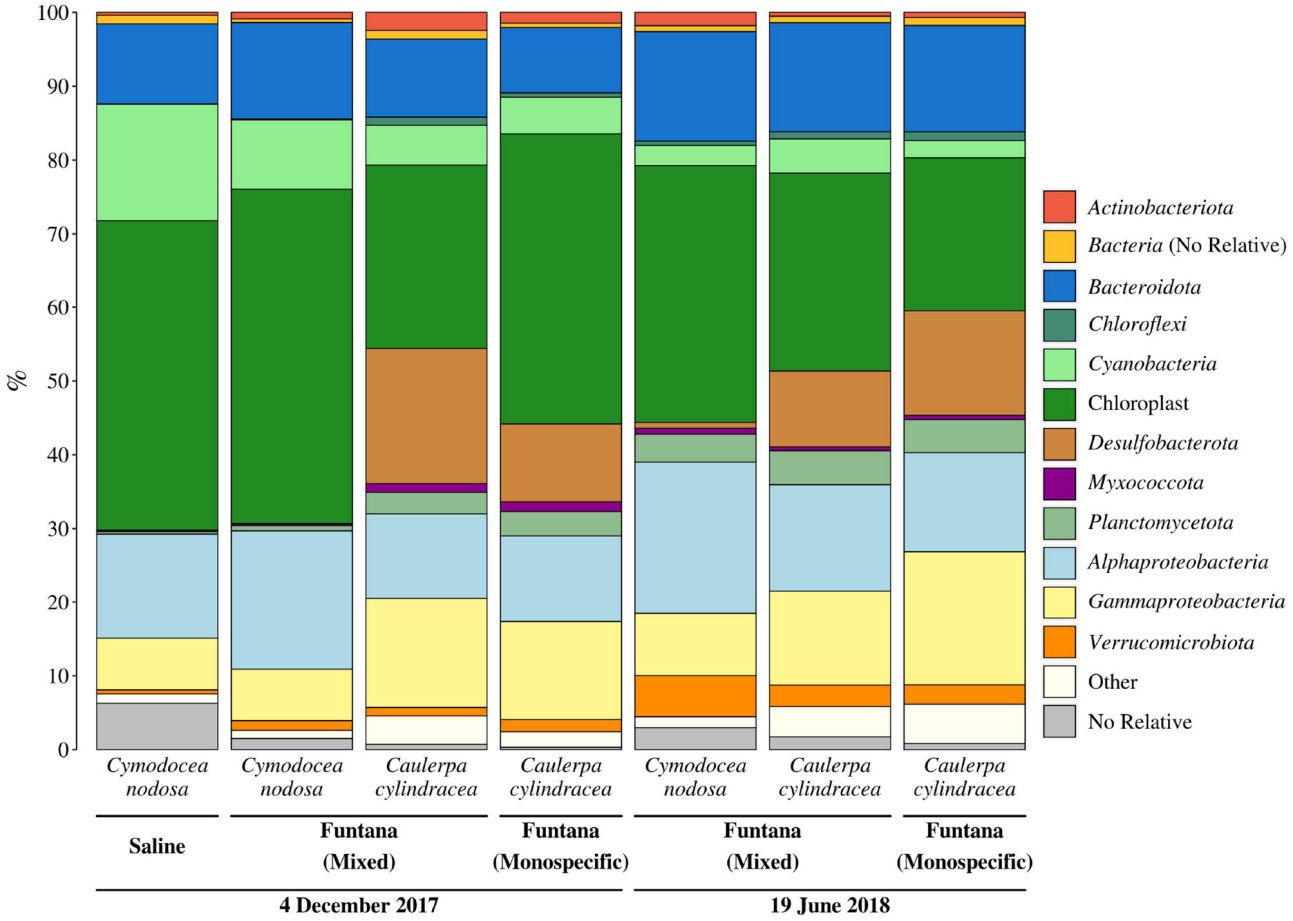
Taxonomic classification and relative contribution of the most abundant (≥1%) bacterial and archaeal sequences from surfaces of two marine macrophytes (*C. nodosa* and *C. cylindracea*) sampled in the Bay of Saline and the Bay of Funtana (mixed and monospecific settlements) and in two contrasting seasons (December 4, 2017 and June 19, 2018).

To evaluate whether the obtained DNA is suitable to determine the composition of the microbial community, Illumina sequencing of the V4 region of the 16S rRNA was performed. Sequencing yielded a total of 292,888 sequences after quality curation and exclusion of eukaryotic, mitochondrial, and no relative sequences. The number of sequences classified as chloroplasts was 97,331. After excluding these sequences, the total number of retrieved reads was 195,557, ranging from 13,667 to 41,842 sequences per sample (Supplementary Table 1). Even when the highest sequencing effort was applied, the rarefaction curves did not level off which is commonly observed in high-throughput 16S rRNA amplicon sequencing (Supplementary Figure 1). Sequences clustering at a similarity level of 97% yielded a total of 8,355 different OTUs. Taxonomic classification of reads revealed a macrophyte-associated epiphytic community mainly composed of *Alphaproteobacteria* (14.9 ± 3.5%), *Bacteroidota* (12.5 ± 2.4%), *Gammaproteobacteria* (11.6 ± 4.3%), *Desulfobacterota* (7.8 ± 7.5%), *Cyanobacteria* (6.5 ± 4.7%), and *Planctomycetota* (2.9 ± 1.7%) (Figure 3).

**Figure 4:**
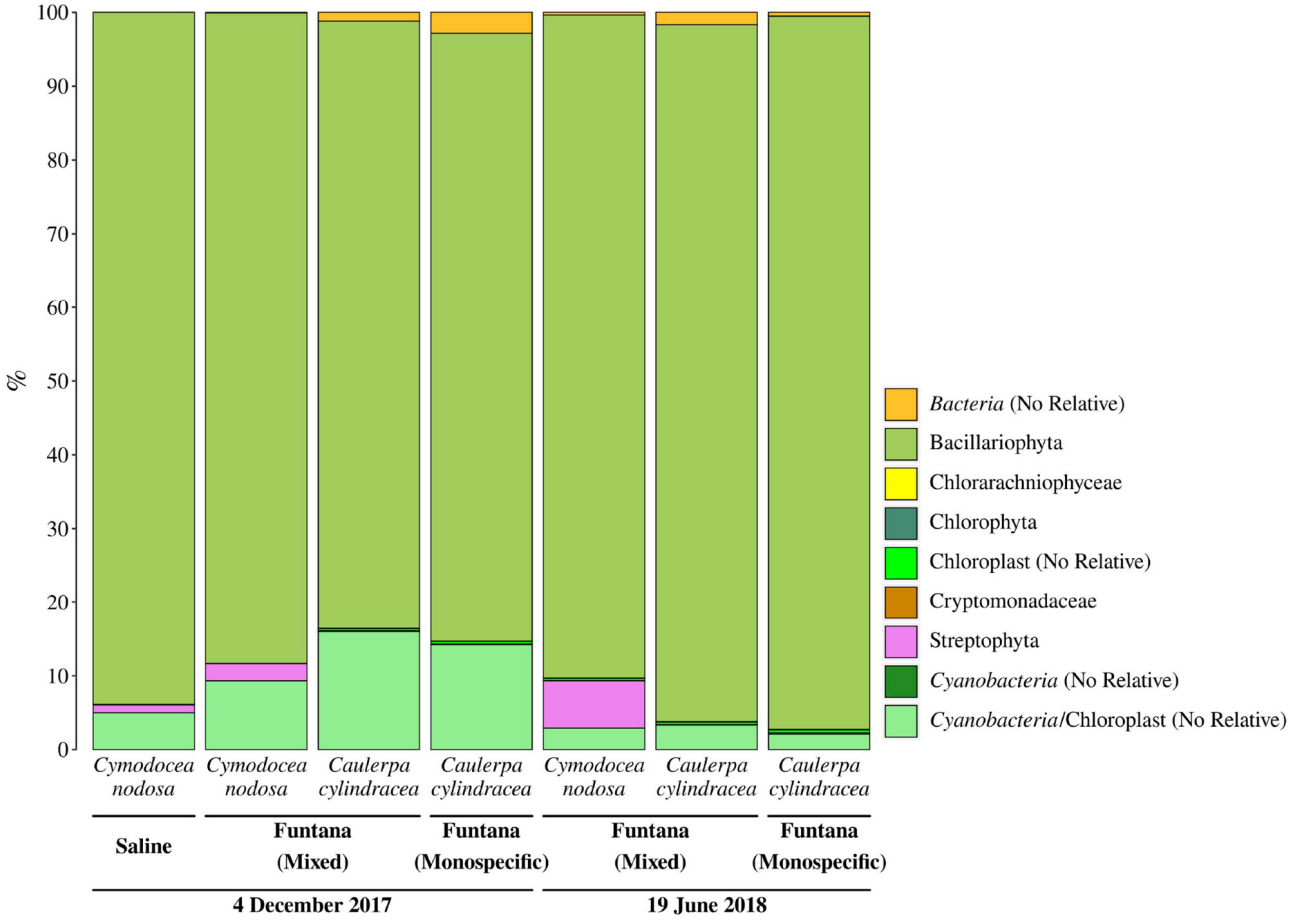
Taxonomic classification and relative contribution of chloroplast sequences from surfaces of two marine macrophytes (*C. nodosa* and *C. cylindracea*) sampled in the Bay of Saline and the Bay of Funtana (mixed and monospecific settlements) and in two contrasting seasons (December 4, 2017 and June 19, 2018).

Primers used in this study, as in many other 16S rRNA amplicon procedures, also amplified chloroplast 16S rRNA genes. We observed a high proportion of chloroplast sequences in all analyzed samples (33.4 ± 9.4%) (Figure 3). To determine whether chloroplast sequences originate from the host or eukaryotic epiphytic organisms, we exported SILVA-classified chloroplast sequences and reclassified them using the RDP training set that allows for a more detailed chloroplast classification. The largest proportion of sequences was classified as Bacillariophyta (89.7 ± 5.7%) indicating that the DNA removal procedure resulted in only minor co-extracted quantities of host DNA (Figure 4). Chloroplast sequences classified as Streptophyta constituted 3.3 ± 2.8% of all chloroplast sequences originating from *C. nodosa* samples, while sequences classified as Chlorophyta comprised only 0.02 ± 0.01% of all chloroplast sequences associated with *C. cylindracea* samples.

**Figure 5:**
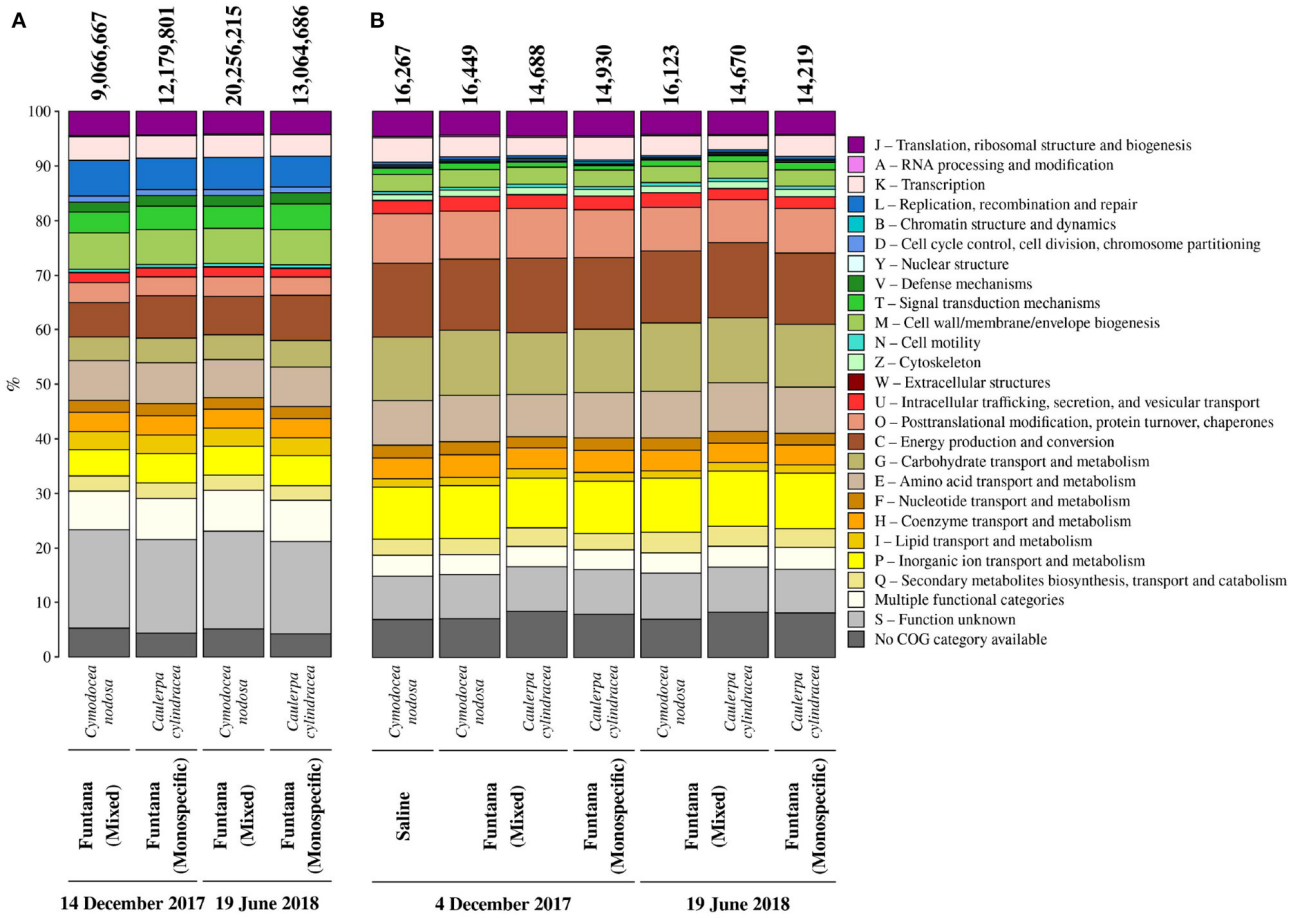
Relative contribution of each COG (Clusters of Orthologous Groups) category to the total number of annotated coding sequences **(A)** from metagenomes and identified proteins **(B)** from metaproteomes associated with surfaces of two marine macrophytes (*C. nodosa* and *C. cylindracea*) sampled in the Bay of Saline and the Bay of Funtana (mixed and monospecific settlements) and in two contrasting seasons (December 4/14, 2017 and June 19, 2018). Total number of annotated coding sequences and identified proteins is given above the corresponding bar.

To determine whether the extracted DNA can be used for metagenomic sequencing, four samples containing epiphytic DNA were selected and shotgun sequenced using an Illumina platform. Metagenomic sequencing yielded between 207,149,524 and 624,029,930 sequence pairs (Supplementary Table 2). Obtained sequences were successfully assembled into contigs whose L50 ranged from 654 to 1,011 bp. In addition, predicted coding sequences were functionally annotated (9,066,667–20,256,215 annotated sequences; Figure 5A) and taxonomically classified. Functional annotation allowed for an assessment of the relative contribution of each COG (Clusters of Orthologous Groups) functional category to the total number of annotated coding sequences (Figure 5A). Functional categories containing the highest number of sequences were C (energy production and conversion), E (amino acid transport and metabolism), M (cell wall/membrane/envelope biogenesis), L (replication, recombination and repair), and P (inorganic ion transport and metabolism). If host DNA is co-extracted with epiphytes, it should be detected in large proportions in sequenced metagenomes. However, no large proportions of coding sequences classified as Streptophyta and Chlorophyta were detected (Supplementary Table 3). Sequenced metagenomic DNA originating from the surface of *C. nodosa* contained 1.3% of coding sequences classified as Streptophyta in December 2017 and 0.7% in June 2018. Furthermore, the summed RPKM (reads per kilobase million) of these sequences constituted 1.7% of total RPKM of all successfully classified sequences in December 2017 and 1.1% in June 2018. Similar low proportions of host coding sequences were detected in metagenomic samples originating from the surfaces of *C. cylindracea*. Of all successfully classified coding sequences, 0.2% sequences were classified as Chlorophyta in December 2017 and 0.1% in June 2018. A relatively higher proportion of RPKM of these sequences than in the case of *C. nodosa* was observed, indicating a higher co-extraction of host DNA in *C. cylindracea*. In December, the proportion of RPKM of sequences classified as Chlorophyta was 8.2%, while in June 2018 it reached 13.6%.

To evaluate whether the procedure for protein extraction is suitable for metaproteomic analysis, obtained proteins were trypsin digested and sequenced using a mass spectrometer. Obtained MS/MS spectra were searched against a protein database from sequenced metagenomes. From 14,219 to 16,449 proteins were identified in isolated protein samples (Figure 5B). In addition, successful identification of proteins allowed for an assessment of the relative contribution of each COG functional category to the total number of identified proteins (Figure 5B). Functional categories containing the highest number of identified proteins were C (energy production and conversion), G (carbohydrate transport and metabolism), P (inorganic ion transport and metabolism), O (post-translational modification, protein turnover, chaperones), and E (amino acid transport and metabolism). Isolated proteins could originate from epiphytic organisms inhabiting the macrophyte surface and/or from macrophyte tissue underlying them. The contribution of proteins originating from host tissues was evaluated by identifying all the proteins predicted to belong to any taxonomic group within the phyla Streptophyta and Chlorophyta and by calculating their contribution to the number and abundance (NAAF [normalized abundance area factor]) of all identified proteins. On average, proteins isolated from the surface of *C. nodosa* contained 1.8 ± 0.06% of proteins associated with Streptophyta, contributing to 2.2 ± 0.8% of total proteins. Similar to metagenomes, proteins associated with Chlorophyta contributed more to *C. cylindracea* than proteins associated with Streptophyta to *C. nodosa*. Chlorophyta-associated proteins composed 5.2 ± 0.06% of all identified proteins in *C. cylindracea*, contributing 19.2 ± 1.5% to the total protein abundance.

## Discussion

To test whether the developed DNA and protein isolation protocols efficiently detach microbes from the macrophyte surface, we selected *C. nodosa* and *C. cylindracea* as representatives of seagrass and macroalgal species. These species differ morphologically. While *C. nodosa* leaves are flat, *C. cylindracea* thalli are characterized by an uneven surface (Kuo and den Hartog, 2001; Verlaque et al., 2003). The developed protocol led to an almost complete removal of epiphytic cells from the surfaces of both species comparable to the result of Burke et al. (2009), indicating that structural differences do not impact the removal efficiency. In addition, isolation protocols were tested in two contrasting seasons, as it is known that macrophytes are harboring more algal epiphytes during autumn and winter (Reyes and Sansón, 2001). No differences in the removal efficiency was observed between seasons, suggesting that these protocols can be used on macrophyte samples retrieved throughout the year. Also, no removal differences were observed on samples derived from the same host but from different locations.

Successful amplification and sequencing of the V4 region of the 16S rRNA gene proved that the isolated DNA can be used to estimate the microbial epiphytic diversity. Taxonomic groups detected in this step can also be often found in epiphytic communities associated with other macrophytes (Burke et al., 2011b; Morrissey et al., 2019). A problem often encountered in studies focusing on epiphytic communities is the presence of large proportions of chloroplast 16S rRNA sequences in the pool of amplified molecules, especially if the epiphytic DNA was isolated without prior selection (Staufenberger et al., 2008). These sequences can derive from host chloroplasts or from eukaryotic epiphytic chloroplast DNA. Although the proportion of obtained chloroplast 16S rRNA sequences in our samples was substantial, they derived almost exclusively from eukaryotic epiphytes. High proportion of chloroplast 16S rRNA sequences in studies applying selective procedures that include direct cellular lysis on host surfaces were observed before (Michelou et al., 2013). It is possible that chloroplast-specific sequences even in these studies originated from eukaryotic epiphytic cells and not from host chloroplasts. Indeed, it is common during 16S rRNA profiling of pelagic microbial communities to observe high proportions of chloroplast sequences (Gilbert et al., 2009; Korlević et al., 2016). One of the solutions to further reduce chloroplast 16S rRNA sequence contamination is to use primers that minimize the amplification of these reads if the sequencing and study design allow it (Hanshew et al., 2013). In addition, a very low proportion of chloroplast 16S rRNA sequences in samples originating from *C. cylindracea* in comparison to *C. nodosa* could be explained by the presence of three introns in the gene for 16S rRNA in some members of the genus *Caulerpa* that could hamper the amplification process (Lam and Lopez-Bautista, 2016).

High-quality DNA is also needed for metagenomics. The obtained number of metagenomic sequences and assembly statistics were comparable to metagenomes and metatranscriptomes derived from similar surface-associated communities (Crump et al., 2018; Cúcio et al., 2018). In addition, functional annotation of predicted coding sequences to COG functional categories showed that the obtained metagenomes can be used to determine the metabolic capacity of surface-associated communities (Leary et al., 2014; Cúcio et al., 2018). The proportion of coding sequences, including their RPKM, originating from *C. nodosa* metagenomes and classified as Streptophyta was low indicating that the isolation procedure was specific for epiphytic cells. DNA samples isolated from the surface of *C. cylindracea* exhibited a low proportion of Chlorophyta coding sequences; however, their RPKM was higher than in the samples originating from *C. nodosa*. One of the reasons for this elevated RPKM of Chlorophyta sequences in *C. cylindracea* could be the differences in the tissue structure between these two host species. While *C. nodosa* leaves are composed of individual cells, the thallus of *C. cylindracea* is, like in other siphonous algal species, composed of a single large multinucleate cell (Coneva and Chitwood, 2015). The absence of individual cells in *C. cylindracea* could cause a leakage of genetic material into the extraction buffer causing an elevated presence of host sequences in the samples for metagenome analyses.

To obtain insight into the metabolic status of uncultivated prokaryotes, a metaproteomic approach is required (Saito et al., 2019). The applied protocol for epiphytic protein isolation followed by a metaproteomic analysis identified between 14,219 and 16,449 proteins, which is higher than previously reported for soils (Chourey et al., 2010; Hultman et al., 2015), seawater (Williams et al., 2012), and biofilms (Leary et al., 2014). The functional annotation of identified proteins into COG functional categories showed that the protein isolation protocol can be used to assess the metabolic status of the epiphytic community (Leary et al., 2014). Similar to the results of the metagenomic analysis, the number and abundance of identified proteins affiliated to Streptophyta in *C. nodosa* samples were low, indicating that the procedure is selective for epiphytic cell proteins. In addition, a higher number and abundance of identified proteins associated with Chlorophyta were observed in *C. cylindracea* samples. The cause of this elevated presence of Chlorophyta-associated proteins can be, similar to the DNA isolation protocol, explained by the absence of individual cells in this siphonous alga (Coneva and Chitwood, 2015).

In conclusion, the developed protocols for DNA and protein isolation from macrophyte surfaces almost completely remove the epiphytic community from both, *C. nodosa* and *C. cylindracea*, in different seasons. Also, the obtained DNA and proteins are suitable for 16S rRNA sequencing, metagenomics and metaproteomics analyses while the obtained material contains low quantities of host DNA and proteins making the protocols specific for epiphytes. Furthermore, the protocols are based on universally available laboratory chemicals hence, making them widely applicable.

## Data Availability Statement

The datasets presented in this study can be found in online repositories. The names of the repositories and accession numbers can be found in the article.

## Author Contributions

MK designed the work with the intellectual contribution from GH and MN, and prepared the manuscript with editorial help from MM, ZZ, GH, and MN. MK, MM, ZZ, and MN performed the sampling and laboratory analysis. MK and ZZ analyzed the data. All authors contributed to the article and approved the final submitted version.

## Conflict of Interest

The authors declare that the research was conducted in the absence of any commercial or financial relationships that could be construed as a potential conflict of interest.
