# Supplementary material for: Selective DNA and Protein Isolation From Marine Macrophyte Surfaces
Source: Front Microbiol. 2021 May 24;12:665999. doi: 10.3389/fmicb.2021.665999 (PMC8180852; doi:10.3389/fmicb.2021.665999)
Supplement: Supplementary file 1 [file Data_Sheet_1.PDF]

Supplementary Material

Selective DNA and Protein Isolation from Marine Macrophyte Surfaces

Marino Korlević[1*], Marsej Markovski[1], Zihao Zhao[2], Gerhard J. Herndl[2,3,4], Mirjana Najdek[1]

1. Center for Marine Research, Ruđer Bošković Institute, Croatia

2. Department of Functional and Evolutionary Ecology, University of Vienna, Austria

3. Department of Marine Microbiology and Biogeochemistry, Royal Netherlands Institute for Sea Research (NIOZ), Utrecht University, The Netherlands

4. Vienna Metabolomics Center, University of Vienna, Austria

*To whom correspondence should be addressed:

Marino Korlević

G. Paliaga 5, 52210 Rovinj, Croatia

Tel.: +385 52 804 768

Fax: +385 52 804 780

e-mail: marino.korlevic@irb.hr

Running title: Selective isolation from macrophyte surfaces

Supplementary figure

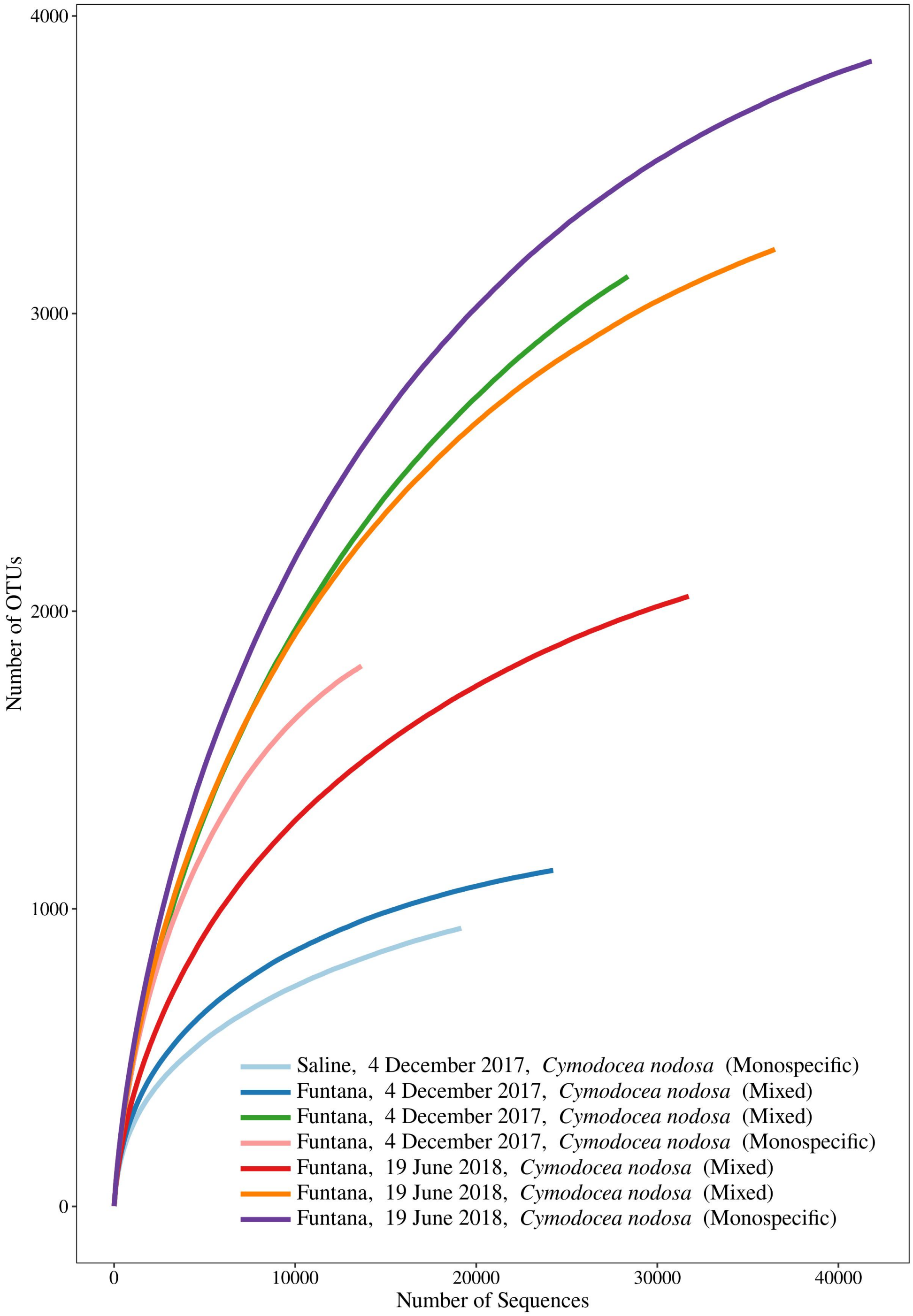

Fig. S1. Rarefaction curves of bacterial and archaeal communities from the surfaces of two marine macrophytes (*C. nodosa* and *C. cylindracea*) sampled in the Bay of Saline and the Bay of Funtana (mixed and monospecific settlements) in two contrasting seasons (4 December 2017 and 19 June 2018).

Supplementary tables

Table S1. Sample ID, sampling station, community type, sampling date, number of sequences and number of OTUs of each sample. The number of sequences and OTUs was calculated after exclusion of sequences without known relatives (no relative sequences) and eukaryotic, chloroplast and mitochondrial sequences.

Sample ID	Station	Community Type	Date	No. of Sequences	No. of OTUs
40	Saline	*Cymodocea nodosa* (Monospecific)	4 December 2017	19,176	935
41	Funtana	*Cymodocea nodosa* (Mixed)	4 December 2017	24,252	1,129
42	Funtana	*Caulerpa cylindracea* (Mixed)	4 December 2017	28,385	3,125
43	Funtana	*Caulerpa cylindracea* (Monospecific)	4 December 2017	13,667	1,816
61	Funtana	*Cymodocea nodosa* (Mixed)	19 June 2018	31,736	2,050
62	Funtana	*Caulerpa cylindracea* (Mixed)	19 June 2018	36,499	3,216
63	Funtana	*Caulerpa cylindracea* (Monospecific)	19 June 2018	41,842	3,848

Table S2. Sample ID, sampling station, community type, sampling date, number of raw sequence pairs, number of assembled contigs by MEGAHIT, N50 and L50 assembly statistics, number of predicted coding sequences (CDS) by Prodigal and number of eggNOG-mapper annotated CDS.

**Sample ID**	**Station**	**Community Type**	**Date**	**No. of Raw Sequence Pairs**	**No. of Contigs**	**N50***	**L50 (bp)***	**No. of Predicted CDS**	**No. of Annotated CDS**
45	Funtana	*Cymodocea nodosa* (Mixed)	14 December 2017	288,446,922	10,786,127	1,814,108	1,011	15,230,601	9,066,667
47	Funtana	*Caulerpa cylindracea* (Monospecific)	14 December 2017	207,149,524	14,541,483	3,417,214	684	19,415,048	12,179,801
61	Funtana	*Cymodocea nodosa* (Mixed)	19 June 2018	624,029,930	25,843,073	5,036,213	873	35,296,634	20,256,215
63	Funtana	*Caulerpa cylindracea* (Monospecific)	19 June 2018	241,132,752	15,909,915	4,071,946	654	20,643,084	13,064,686

* The notation was preserved from the original output of BBTools stats.sh.

Table S3. Phyla into which CDS were classified, number and proportion of CDS in different phyla and sum of coding sequences' RPKM (Reads Per Kilobase Million) and their proportion in different phyla. Data are derived from sequenced metagenomes. Each metagenomic sample is labelled with sampling station, community type, sampling date and sample ID. For each sample top ten phyla based on RPKM were selected. CDS that were not successfully classified were excluded from the dataset.

Phylum	No. of CDS	CDS (%)	Summed RPKM	RPKM (%)
**Funtana, *Cymodocea nodosa* (Mixed), 14 December 2017 (sample ID: 45)**				
Proteobacteria	4,021,486	57.08	526,874.683	56.71
Cyanobacteria	459,942	6.53	155,294.303	16.72
Bacteroidetes	1,388,758	19.71	116,972.727	12.59
Actinobacteria	298,264	4.23	38,176.240	4.11
Bacillariophyta	252,243	3.58	33,816.874	3.64
**Streptophyta**	91,316	1.30	15,839.692	1.71
Rhodophyta	76,937	1.09	15,298.496	1.65
Planctomycetes	227,863	3.23	9,778.230	1.05
Verrucomicrobia	39,912	0.57	2,142.129	0.23
Chloroflexi	35,228	0.50	1,848.597	0.20
**Funtana, *Caulerpa cylindracea* (Monospecific), 14 December 2017 (sample ID: 47)**				
Proteobacteria	5,384,137	61.24	493,882.897	57.93
Bacteroidetes	1,187,188	13.50	77,090.557	9.04
**Chlorophyta**	13,745	0.16	70,249.251	8.24
Actinobacteria	444,926	5.06	55,212.308	6.48
Cyanobacteria	363,766	4.14	41,167.514	4.83
Planctomycetes	502,248	5.71	27,522.230	3.23
Bacillariophyta	243,702	2.77	23,336.154	2.74
Verrucomicrobia	178,424	2.03	10,152.046	1.19
Porifera	17,398	0.20	10,105.655	1.19
Rhodophyta	48,544	0.55	7,117.314	0.83
**Funtana, *Cymodocea nodosa* (Mixed), 19 June 2018 (sample ID: 61)**				
Proteobacteria	8,185,781	55.21	573,484.714	65.33
Bacteroidetes	2,226,547	15.02	78,921.488	8.99
Bacillariophyta	761,510	5.14	71,003.503	8.09

Table S3. Phyla into which CDS were classified, number and proportion of CDS in different phyla and sum of coding sequences' RPKM (Reads Per Kilobase Million) and their proportion in different phyla. Data are derived from sequenced metagenomes. Each metagenomic sample is labelled with sampling station, community type, sampling date and sample ID. For each sample top ten phyla based on RPKM were selected. CDS that were not successfully classified were excluded from the dataset. *(continued)*

Phylum	No. of CDS	CDS (%)	Summed RPKM	RPKM (%)
Actinobacteria	655,055	4.42	43,153.463	4.92
Planctomycetes	1,338,538	9.03	35,240.242	4.01
Cyanobacteria	474,226	3.20	24,941.374	2.84
Verrucomicrobia	371,837	2.51	19,794.186	2.25
**Streptophyta**	108,235	0.73	9,555.978	1.09
Chloroflexi	150,402	1.01	3,438.376	0.39
Mollusca	50,750	0.34	1,768.748	0.20
**Funtana, *Caulerpa cylindracea* (Monospecific), 19 June 2018 (sample ID: 63)**				
Proteobacteria	5,429,374	60.24	440,876.241	56.73
**Chlorophyta**	13,360	0.15	105,595.034	13.59
Bacteroidetes	1,084,784	12.04	64,827.936	8.34
Planctomycetes	794,696	8.82	43,962.118	5.66
Actinobacteria	394,004	4.37	28,048.256	3.61
Cyanobacteria	237,026	2.63	17,046.410	2.19
Verrucomicrobia	203,253	2.26	12,033.107	1.55
Bacillariophyta	174,954	1.94	11,959.067	1.54
Chloroflexi	175,514	1.95	8,952.527	1.15
**Streptophyta**	4,771	0.05	6,752.150	0.87
